# Bufadienolide Penetration Through the Skin Membrane and Antiaging Properties of *Kalanchoe* spp. Juices in Dermal Applications

**DOI:** 10.3390/molecules30040802

**Published:** 2025-02-09

**Authors:** Anna Hering, Krzysztof Cal, Mariusz Kowalczyk, Alina Kastsevich, Yahor Ivashchanka, J. Renata Ochocka, Justyna Stefanowicz-Hajduk

**Affiliations:** 1 Department of Biology and Pharmaceutical Botany, Medical University of Gdańsk, Al. Gen. J. Hallera 107, 80-416 Gdańsk, Poland; jadwiga.ochocka@gumed.edu.pl; 2 Department of Pharmaceutical Technology, Medical University of Gdańsk, Al. Gen. J. Hallera 107, 80-416 Gdańsk, Poland; krzysztof.cal@gumed.edu.pl; 3 Department of Biochemistry and Crop Quality, Institute of Soil Science and Plant Cultivation, State Research Institute, 24-100 Puławy, Poland; mkowalczyk@iung.pulawy.pl; 4 Faculty of Pharmacy, Medical University of Gdańsk, Al. Gen. J. Hallera 107, 80-416 Gdańsk, Poland; kostevichalina@gumed.edu.pl (A.K.); egorivashka@gumed.edu.pl (Y.I.)

**Keywords:** bufadienolide, skin aging, anti-inflammatory properties, whitening effect, *Kalanchoe*, free radicals, Strat-M membrane

## Abstract

Skin aging is accelerated by inflammation processes generated by oxidative stress and external factors such as UV radiation. Plants belonging to the genus *Kalanchoe* that are rich sources of antioxidants could potentially strengthen the skin barrier if used as ingredients in cosmetic formulations. However, their use is limited due to the contents of bufadienolides, known cardiotoxins. This study aimed to establish a semi-quantitative profile of bufadienolides in the juices of *K. blossfeldiana*, *K. daigremontiana*, and *K. pinnata* using UHPLC combined with charged aerosol detection (CAD) and high-resolution mass spectrometry (HR-MS). Additionally, the study determined the ability of bufadienolides to penetrate the skin barrier using the Bronaugh Diffusion Cell Apparatus and Strat-M membrane. The study also assessed the ferric and molybdenum-reducing powers, as well as the radical scavenging capabilities of these plants juices using 2,2-diphenyl-1-picrylhydrazyl (DPPH) and 2,2-azinobis-(3-ethylbenzothiazoline-6-sulfonate) (ABTS) methods. The in vitro antihyaluronidase and antityrosinase activities and sun protection factor (SPF) were evaluated spectrophotometrically, indicating moderate capability to inhibit the skin enzymes, but low SPF protection for all analyzed juices. The semi-qualitative analysis demonstrated the presence of bufadienolides occurring in two juices from *K. daigremontiana* and *K. pinnata*, with the highest contents of 1,3,5-bersaldegenin-orthoacetate, bryophyllin-A/bryotoxin-C, bersaldegenin-acetate/bryophyllin-C, and diagremontianin. After passing through the skin model, no bufadienolide compounds were present in the subcutaneous filtrate. Antiradical and reduction assays revealed the antioxidant potential of *K. blossfeldiana* and *K. pinnata*. These results indicate that *Kalanchoe* juices have antiaging potential and appear safe for dermal applications.

## 1. Introduction

Skin aging includes several processes related to the deterioration of the natural functions of individual skin layers. The blood supply and hydration of the skin decrease with age, significantly limiting the regeneration processes. Macromolecules responsible for maintaining the structure are destroyed, and the process of melanin synthesis is also disrupted. Additionally, a low-quality diet, smoking, exposure to UV radiation, and constant stress deepen the inflammatory processes accompanying skin aging. An additional element affecting the skin’s condition is exposure to external free radicals and increased internal free radical generation. Both are responsible for accelerating the degradation and inflammatory processes within the skin layers [1,2]. The current cosmetic market strives to search for natural ingredients that can fight free radicals, slow down the processes that degrade skin macromolecules, and reduce excessive pigmentation. Plant juices or extracts deliver to the skin many vital nutrients that not only support regeneration processes, but also nourish the skin and protect it against bacterial and fungal infections [3].

Some synthetic and some natural, commonly used constituents of antiaging preparations may cause diverse side effects, like inflammation or allergy [4]. An example is kojic acid, commonly used for the active inhibition of tyrosinase and pigmentation disorders. The compound protects the skin from UV radiation and extends the shelf life of cosmetic products [5]. Adding plant-originated ingredients to the formulation can significantly improve the protective properties of cosmetic preparations [3]. Species from the *Kalanchoe* genus may be a potential source of compounds that improve skin functions and limit inflammation processes [6]. According to our previously reported research [7,8,9], *K. blossfeldiana*, *K. daigremontiana*, and *K. pinnata* have antimicrobial and antioxidant properties, while the high content of both polyphenol and bufadienolide compounds may also be responsible for many yet undiscovered applications of these plants. *Kalanchoe pinnata* is one of the most extensively analyzed species with high anti-inflammatory and wound-healing properties [6,10,11]; however, many molecular aspects of its action have not been fully elucidated.

The limitation the *Kalanchoe* plants use is the high content of bufadienolide compounds, known for their cardiotoxic effects [8,9,12,13]. This study aimed to qualitatively and semi-quantitatively determine the composition of bufadienolides in *Kalanchoe* spp. juices and, for the first time, assess the ability of these compounds to penetrate the skin. Stratum corneum is the main barrier of the skin, and according to Lipinski et al., to pass this layer, the compound should have specific features: a weight of less than 500 Da and Log P between 1 to 3. Other limitations are the branched structure and number of hydrogen donors and acceptors [14]. According to those data, bufadienolides should not be capable of penetrating through the skin, although plant extracts can influence the skin barrier and alter skin permeability [15]. To test semi-solid and liquid preparations for skin permeation, the Organization for Economic Co-operation and Development (OECD) and European Medicines Agency (EMA) allow the use of human or animal skin [16,17,18]. However, this type of research is limited for ethical reasons, among others. In recent years, synthetic membranes have become a standard, which is beneficial primarily due to the elimination of the significant variability of results obtained from the specificity of natural membranes obtained for ex vivo tests. Many comparative ex vivo studies of human and animal skin with synthetic membranes have been conducted. The results allow to evaluate the diffusivity of compounds and even confirm the possibility of obtaining a satisfactory correlation between both experimental models. The permeation methodology is consistent with OECD and EMA guidelines [16,17,18]. The Strat-M membrane (Merck Millipore, Burlington, MA, USA) was chosen as the synthetic membrane whose specific two-layer structure (polyolefins and polysulfoethers) imitates the epidermis and dermis layers. Currently, this membrane is considered the most suitable synthetic substitute for human skin for testing the penetration and permeation rate of both active and auxiliary substances through the skin [19,20,21].

In this work, we evaluate the potential of the obtained juices from *K. blossfeldiana*, *K. daigremontiana*, and *K. pinnata* in antiaging cosmetic preparations, in terms of antioxidant and reducing activity, as well as the ability to protect from UV radiation and the inhibition of extracellular enzymes (ECM) responsible for skin discoloration, dryness, and inflammation processes. A significant aspect of the work is the assessment, for the first time, of whether bufadienolide compounds can penetrate skin membranes. The present research is the first step in determining if the juices of *K. blossfeldiana*, *K. daigremontiana*, and *K. pinnata*, expressing antiaging potential, can be used in skin-care products.

## 2. Results

### 2.1. Qualitative and Semi-Quantitative Analysis of Bufadienolides Content

Approximately 40 bufadienolides (C-24 steroids with an α-pyrone ring attached at the C-17β position) have been identified in the species belonging to the genus *Kalanchoe* [22]. Although there are more than 170 species of *Kalanchoe* worldwide, bufadienolides were formally confirmed to occur in only five of them, including *K. daigremontiana* and *K. pinnata*. Bufadienolides were not previously reported from *K. blossfeldiana* or detected in the juice used in this study [7]. All detected bufadienolides were present as free steroids, while steroid glycosides were not observed.

The literature indicates that the highest number of bufadienolides was identified in *K. daigremontiana* [22]. Our results are similar—*K. daigremontiana* juice had the highest contents and widest variety of the detected bufadienolides (Table 1). The most abundant bufadienolide (estimated by LC-CAD contents of 80.4 mg/100 g d.w.) of *K. daigremontiana* juice had RT 13.9 min and produced [M+H]^+^ ion at *m*/*z* 457.22, corresponding to molecular formula C_26_H_32_O_7_. The two significant neutral losses, 78.04 Da and 104.05 Da, occurring due to the loss of orthoacetate and carbonyl function resulting in the ring A opening, suggested the potential identity of this peak as bersaldegenin orthoacetate. The identity was subsequently confirmed using the authentic standard of bersaldegenin-1,3,5-orthoacetate. The same diagnostic neutral losses were observed for the peak with RT 8.3 min, indicating it also possesses the orthoacetate function. However, the observed precursor ion was at *m*/*z* 487.196, corresponding to C_26_H_30_O_9_, suggesting the presence of additional hydroxyl and carbonyl groups. Therefore, the structure of this peak could match daigremontianin, which, again, was confirmed with the reference standard obtained previously [23]. Another peak with fragmentation spectrum showing losses of 78.04 and 104.05 had RT at 4.9 min and the precursor ion at *m*/*z* 473.22, differing from bersaldegenin orthoacetate by 16 Da. A comparison with authentic standards indicated that it represents bryophyllin A. Similar neutral losses were also observed for the peak at RT 4.8 min, containing a component with protonated ion at *m*/*z* 489.21. Compared to bryophyllin A, the calculated formula of the precursor ion (C_26_H_32_O_8_) indicated two additional hydrogens. Therefore, this component likely represents bryotoxin B.

The RT 3.7 and 5.6 min peaks produced identical, superimposable MS2 spectra and identical precursor ions at *m*/*z* 475.23. The diagnostic ion at *m*/*z* 415.210 (the loss of 60.02 Da) indicated the presence of acetate. Therefore, the two molecules likely represent bersaldegenin acetates. However, establishing the esterification position was impossible because of the similarities between the fragmentation spectra. The identity of these two compounds was not fully confirmed due to the lack of authenticated reference standards. The related compound with a peak at RT 2.7 min produced similar fragmentation spectra, but had its protonated precursor ion at *m*/*z* 491.227, corresponding to the ion formula C_26_H_34_O_9_. An additional oxygen atom, as well as identical to the two previously described compounds’ count of hydrogens, both indicate an additional hydroxyl group. It may suggest that the compound is bryophyllin B, but the identification was not confirmed due to the lack of an appropriate standard.

As expected, based on the literature data [24], the *K. pinnata* juice profile did not contain daigremontianin and contained only minor amounts of bersaldegenin acetate and orthoacetate.

Generally, the contents of bufadienolides in the investigated juices were relatively low, and the levels of individual compounds were similar to those reported previously [24]. The MS spectra of identified bufadienolide compounds with their collision energy of compounds B–L are included in Supplementary Materials, Figure S1A–F.

**Table 1:** Bufadienolides identified and semi-quantified in tested juices of leaves from different species of *Kalanchoe* L. using UHPLC-CAD-QTOF analysis [mg/100 g of the dry weight].

	Name	Formula	RT [min]	Expected [M+H]^+^ *m*/*z*	Measurement Error [mDa]	Isotopic Fit (mσ)	Identification Conf. Level	*K. daigremontiana*	*K. pinnata*
A	3β -(O-α-L-rhamnopyranosyl)-5 β,11α,14 β,19-tetrahydroxybufa-20,22-diene (ref. cmpd.)	C_30_H_44_O_11_	2.1	581.2956	0.7	59.2	1	trace	n.d.
B	bryophyllin B	C_26_H_34_O_9_	2.7	491.2276	0.2	10.4	2	trace	trace
C	tetrahydroxy-bufadiene-O-dHex (an isomer of 1)	C_30_H_44_O_11_	2.9	581.2956	−0.1	24.5	2	trace	n.d.
D	bersaldegenin-acetate isomer 1	C_26_H_34_O_8_	3.7	475.2326	0.5	78.5	2	trace	2.6 ± 0.7
E	bryotoxin-B	C_26_H_32_O_9_	4.8	489.2119	0.2	5.3	3	trace	n.d.
F	bryophyllin A/bryotoxin C (ref.cmpd.)	C_26_H_32_O_8_	4.9	473.2174	−0.2	21.0	1	5.9 ± 0.5	n.d.
G	bersaldegenin-acetate isomer 2	C_26_H_34_O_8_	5.6	475.2326	−0.1	8.5	3	trace	trace
H	bryophyllin A/bryotoxin C isomer 2	C_26_H_32_O_8_	5.7	473.2176	−0.8	22.4	3	trace	n.d.
I	diagremontianin isomer	C_26_H_30_O_9_	6.2	487.1963	−0.9	24.5	3	1.9 ± 0.4	n.d.
J	bryophyllin -C	C_26_H_34_O_8_	6.9	475.2326	−0.7	7.2	2	3.0 ± 0.4	trace
K	diagremontianin (ref. cmpd.)	C_26_H_30_O_9_	8.3	487.1963	−0.5	9.2	1	4.6 ± 0.4	n.d.
L	1,3,5-bersaldegenin-orthoacetate (ref. cmpd.)	C_26_H_32_O_7_	13.9	457.2221	0.7	4.2	1	80.4 ± 1.0	7.7 ± 0.6

n.d.—not detected; trace—contents below the lower limit of detection (<0.2 mg/100 g dry weight); dHex—deoxyhexose, contents presented as mean value ± SD (*n* = 3), identification confidence levels as described in Schymanski et al. [25]; R.T—retention time; ref.cmpd—reference compound; *K. daigremontiana*—*Kalanchoe daigremontiana*; *K. pinnata*—*Kalanchoe pinnata*.

### 2.2. Bufadienolides’ Permeability Through the Strat-M Membrane

**Figure 1:**
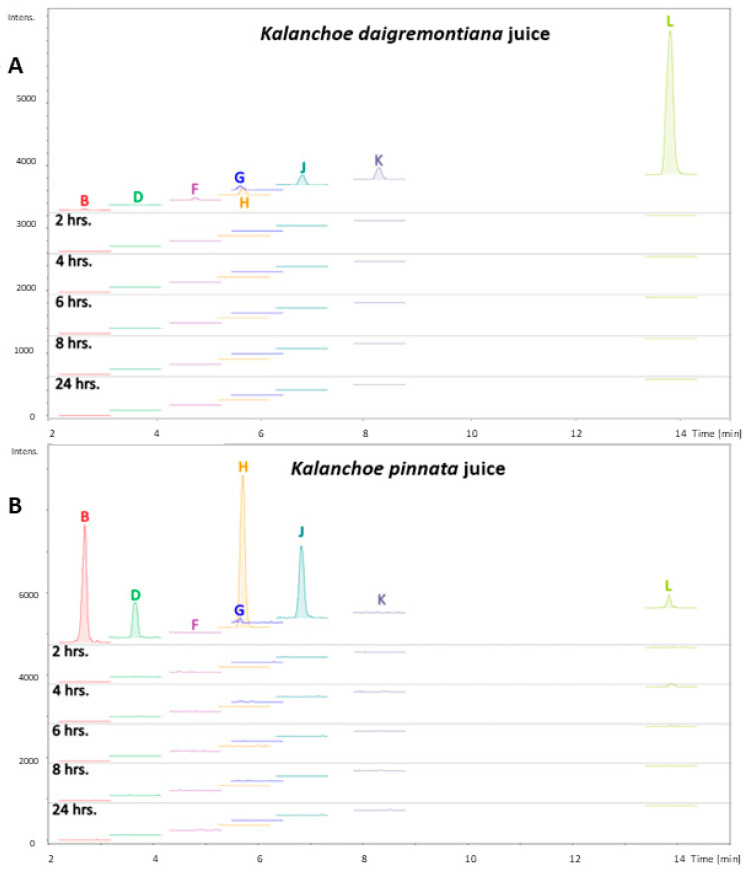
Extracted ion chromatograms (precursor ion *m*/*z* ± 0.002 ppm) of bufadienolides from the *Kalanchoe daigremontiana* (**A**) and *Kalanchoe pinnata* (**B**) juices and Strat-M membrane filtrates after 2, 4, 6, 8, and 24 hrs (B: bryophyllin B, D: bersaldegenin acetate isomer, F: bryophyllin A, G: bersaldegenin acetate isomer, H: bryophyllin A isomer, J: bryophyllin C, K: daigremontanin; L: bersaldegenin orthoacetate).

The Strat-M membrane was utilized to assess the transdermal diffusion of the tested bufadienolides listed in Table 1. The membrane was placed in the flow-through Bronaugh diffusion cell apparatus, and juices of *K. blossfeldiana*, *K. daigremontiana*, and *K. pinnata* were applied on the surface of the Strat-M membrane. In the juice of *K. blossfeldiana*, bufadienolides were not detected. Figure 1 shows the ion chromatograms of bufadienolides identified in the juices of *K. daigremontiana* (Figure 1A) and *K. pinnata* (Figure 1B) marked in colors (B: bryophyllin B, D: bersaldegenin acetate isomer, F: bryophyllin A, G: bersaldegenin acetate isomer, H: bryophyllin A isomer, J: bryophyllin C, K: daigremontanin; L: bersaldegenin orthoacetate). The filtrates were collected after 2, 4, 6, 8, and 24 h from application. The qualitative and quantitative studies of the collected filtrates of the acceptor fluid did not show the presence of bufadienolide compounds in any of them, even after 24 h of the experiments.

### 2.3. Antioxidant Activity

To determine the scavenging free radicals, as well as reducing properties of the juices obtained from the leaves of *K. blossfeldiana*, *K. daigremontiana*, and *K. pinnata*, the DPPH, ABTS, FRAP (ferric reducing power), and molybdenum reducing power assays were performed. In all four analyses, ascorbic acid was used as a standard.

**Figure 2:**
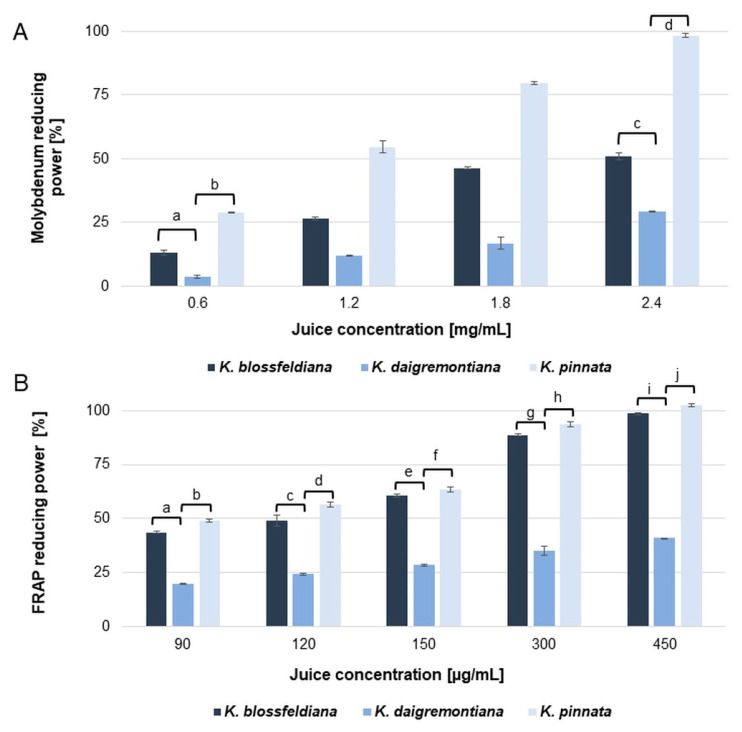
Molybdenum reducing power (**A**) and ferric reducing power (**B**) of *Kalanchoe* spp. juices [%]. The results were obtained from three independent experiments with three repetitions (*n* = 9). Mean ± SD. The statistically significant differences among groups are marked with a–j (one-way ANOVA with Tuckey’s post hoc test, *p* < 0.05).

The results revealed moderate, dose-dependent antiradical and reducing capabilities of the tested juices. The resulting IC_50_ values are summarized in Table 2. Among the analyzed juices, the best capability to scavenge free radicals was indicated for *K. blossfeldiana* (50.7 ± 0.77 and 18.8 ± 0.21 µg/mL for the DPPH and ABTS assays, respectively), while only slightly weaker properties were demonstrated for *K. pinnata* (61.1 ± 0.89 and 28.27 ± 0.73 µg/mL for the DPPH and ABTS assays, respectively). However, in reduction tests, better results were observed for juice obtained from *K. pinnata* than *K. blossfeldiana*, as shown in Table 2 and Figure 2. It is necessary to emphasize that juices from both species showed quite good iron-reducing abilities and were significantly weaker in molybdenum reduction compared to the standard ascorbic acid. In the FRAP test, at the 450 µg/mL concentration, *K. pinnata* and *K. blossfeldiana* showed an almost complete iron reduction. In contrast, in the case of molybdenum reduction at the maximum concentration of 2.4 mg/mL, only *K. pinnata* was capable of causing complete reduction. *K. blossfeldiana* juice applied at the same concentration (Figure 2) resulted in 50.93 ± 0.87% of molybdenum reduction power (a further increase of the juice concentration resulted in precipitate formation in the reaction mixture).

Juice from *K. daigremontiana* showed poor free radical scavenging and reduction properties, obtaining IC_50_ at concentration range of 496.13–561.36 in the DPPH, ABTS, and FRAP assays, respectively (Table 2). In the molybdenum reduction test, *K. daigremontiana* juice showed only slight activity and was much weaker than the *K. blossfeldiana* and *K. pinnata* juices. At the highest concentration used in this test, 2.4 mg/mL, *K. daigremontiana* juice could reduce only 29.2% of molybdenum.

### 2.4. Enzyme Inhibition

Tyrosinase is a crucial enzyme in melanin biosynthesis, and its over-reactive activity leads to local discoloration. This unwanted process increases with age, especially in the post-menopausal period [26,27]. Hyaluronic acid is highly involved in proper skin hydration and moisture, while hyaluronidase causes its degradation and also generates inflammation processes [28,29].

The three tested juices of *Kalanchoe* spp. were investigated for antityrosinase and antihyaluronidase capability. The experiments revealed dose-dependent inhibition properties of all three juices on both enzymes. The results expressed as IC_50_ values of *K. blossfeldiana*, *K. daigremontiana*, and *K. pinnata* leaf juices are shown in Table 3.

The influence of the plants’ leaf juices on tyrosinase activity was investigated using L-DOPA as the substrate and kojic acid as a control. With the increase of the juice concentration, tyrosinase activity decreased significantly, although the inhibition was less potent than for the standard substance in all tested juices. The highest influence on the enzyme was detected for *K. pinnata* juice (IC_50_ was 206.13 ± 7.4 µg/mL), while *K. blossfeldiana*, followed by *K. daigremontiana,* expressed lower capability to inhibit tyrosinase (IC_50_ were 317.17 ± 6.94 and 457.12 ± 5.33 µg/mL, respectively).

Similar results were revealed for the antihyaluronidase activity of the leaf juices. We observed that with the increase of the juice concentration, hyaluronidase activity decreased significantly, although the inhibition was less potent than the standard substance—oleanolic acid. Also, in the antihyaluronidase tests, the highest influence on the enzyme was detected for *K. pinnata* juice (IC_50_ was 116.15 ± 5.15 µg/mL), while *K. blossfeldiana* and *K. daigremontiana* expressed two and four times less capability to inhibit hyaluronidase, respectively (Table 3).

### 2.5. The Sun Protection Factor In Vitro of the Kalanchoe spp. Juices

The Sun Protection Factor was estimated spectrophotometrically for the juices of the *Kalanchoe* species. The assumption of this analysis was to assess the ability of the tested juices to protect skin macromolecules against the degradative effects of UV A and UV B radiation.

**Figure 3:**
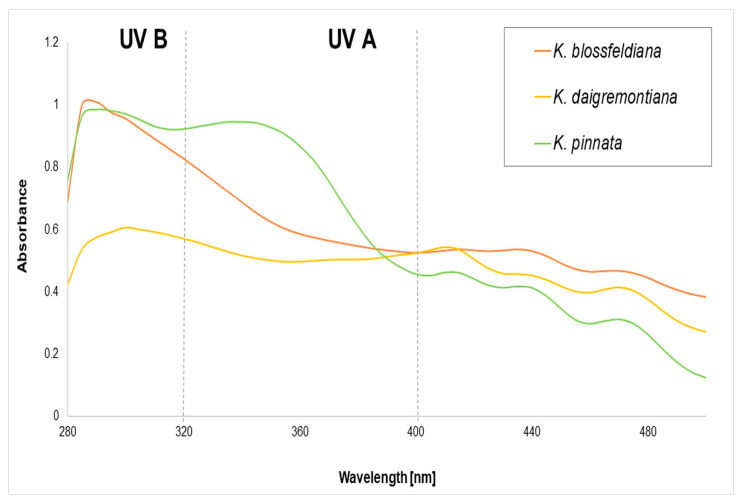
UV absorbance of juices from *K. blossfeldiana*, *K. daigremontiana*, and *K. pinnata* at the concentration of 1 mg/mL (methanol dilution).

A series of dilutions (62.5–1000 µg/mL) of juices from *K. blossfeldiana*, *K. daigremontiana*, and *K. pinnata* were determined for UV spectra (λ 280–500 nm). The obtained UV spectra of the analyzed juices at a concentration of 1 mg/mL are presented in Figure 3. The results revealed wavelength-absorbance changes dependent on the juice. The absorbance levels at the highest used concentration were relatively low compared to single polyphenolic compounds [30]. The highest absorbance range of 280–380 nm was revealed for *K. pinnata* juice, indicating potential protection against UV A (320–400 nm) and UV B (280–320 nm). Juice from *K. blossfeldiana* presented potential protection against UV B, similar to juice from *K. pinnata*, while relatively low against UV A. *K. daigremontiana* exhibited the lowest protection in both the UV A and UV B spectrum.

The calculated sun photoprotection factors of the juices are presented in Table 4 and classified according to the following European Commission recommendations.

- No protection SPF in vitro ≤ 5.9;
- Low protection 6.0 ≤ SPF in vitro ≤ 14.9;
- Medium protection 15.0 ≤ SPF in vitro ≤ 29.9;
- High protection 30.0 ≤ SPF in vitro ≤ 59.9 [31].

The in vitro Sun Protection Factor estimated for juices from the leaves of *K. blossfeldiana*, *K. daigremontiana*, and *K. pinnata* indicated their low capability of general protection. However, only *K. pinnata* juice has potential to protect from both UV A and UV B, while the SPF of *K. daigremontiana* juice offered almost no protection.

## 3. Discussion

Today, facial care products should do more than just correctly moisturize the skin. The skin of the face and hands is the most exposed to oxidative stress caused by external environmental factors. Smog, cigarette smoke, and UV radiation significantly degrade skin macromolecules. With prolonged exposure, extracellular matrix enzymes (ECMs) are excessively stimulated, and collagen, elastin, and hyaluronic acid bonds are destroyed. Internal degradation processes are reflected in externally visible excessive skin dryness, crow’s feet, wrinkles, uneven coloring, and rough skin texture. For these reasons, modern cosmetics should prevent unwanted skin aging [1,2].

Crude leaf juices from *Kalanchoe* L. demonstrated moderate antityrosinase and antihyaluronidase activity. Interestingly, in both enzymatic tests, the strongest inhibition was presented for *K. pinnata*, followed by *K. blossfeldiana* and *K. daigremontiana*. The obtained results are most likely related to the content of polyphenolic compounds in the extracted juices. Both *K. pinnata* and *K. blossfeldiana* leaves are rich sources of these compounds [9,13]. However, most of the analyses conducted so far have been performed on extracts [32,33], while juices, commonly used in ethnomedicine, have not been studied in this field. Plants of the *Kalanchoe* species were analyzed in that work to confirm their utility to treat pathological conditions. As inflammation often leads to the development of disease changes in the body, many studies have focused on the use of plants of the *Kalanchoe* genus in combating inflammation with good results [6,13,22]. However, no direct effect of these kinds of plant juices on hyaluronidase activity has been demonstrated so far. It is well known that inflammatory processes are connected with hyaluronidase activity. These extracellular matrix (ECM) enzymes degrade high molecular weight hyaluronic acid to low molecular weight hyaluronic acid displaying pro-inflammatory properties, such as increasing the expression of cytokines, growth factors, and macrophage activation [28,34,35]. Our results indicated dose-dependent inhibition of this ECM enzyme by the juices from all three plants, with the highest activity in *K. pinnata*. Antihyaluronidase properties of the tested juices may limit skin dryness and visible skin aging processes. The other tested enzyme, tyrosinase, also plays a significant role in skin conditions, and its inhibition may prevent unwanted discoloration. According to the literature, only extracts from *K. thyrsiflora* have indicated a strong antityrosinase activity similar to kojic acid [36].

To keep the skin in good condition, the antioxidant properties of plant extracts and metabolites are commonly utilized. In this work, the best properties useful in reducing free radicals were shown by *K. pinnata* and *K. blossfeldiana* juices. In the previous study, water extracts prepared from *K. blossfeldiana* had high antioxidant activity with IC_50_ values below 10 µg/mL [9]. In other works, ethanol extract from *K. pinnata* exerted antioxidative properties at the IC_50_ of 90 µg/mL [37]. These effects are the result of the presence of, among others, polyphenols in the *Kalanchoe* species [13,33,37]. Other important metabolites in these plants are bufadienolide compounds, which cause an increase in sodium excretion and hypertension, and act as cardiac inotropes [38]. However, excessive levels of these compounds in the body are toxic. Thus, in this study, we estimated the amount of detected bufadienolide metabolites in the tested plant juices and assessed the permeability of these compounds through the membrane skin.

Our quantitative analysis showed that in two juices from *K. daigremontiana* and *K. pinnata* leaves, one compound, bersaldegenin-1,3,5-orthoacetate, predominates, while the other bufadienolides remain in much lower concentrations. It should be emphasized that in the *K. daigremontiana* juice, the concentration of this compound was more than ten times higher than in *K. pinnata* juice. In *K. blossfeldiana* juice, we did not observe bufadienolide compounds.

Further studies on the penetration of bufadienolide compounds through the skin membrane demonstrated no bufadienolides in any of the permeants, even after 24 h. It can be concluded that bufadienolides identified in *K. daigremontiana* and *K. pinnata*, in accordance with Lipinski’s rule [14], are not able to penetrate membranes. The branched structure and a small amount of hydroxyl groups are probably the main factors inhibiting the penetration of these compounds through the skin [12]. It should be noted, however, that the above studies were conducted on juices, where the active compounds occur in a relatively lower concentration. Analogous studies of the penetration of this group of compounds should also be conducted on extracts and/or fractions containing significant amounts of bufadienolide compounds. An additional aspect requiring further analysis is the great diversity of the discussed group of active compounds, where a steroidal aglycone is the main structure that forms a wild range of glycosides in plant and animal kingdoms [12]. For example, in toads, bufadienolides are secreted on the skin to not only protect against pathogens, but also exhibit antitumor and analgesic properties [39,40,41,42]. Among plant families, *Hyacinthaceae* and *Crassulaceae* show the highest amount and diversity of bufadienolide compounds [23,24,43,44,45,46]. Those active constituents are tested for analgesic, antimicrobial, insecticidal, antiviral, antiplasmodial, antihypertensive, and cytotoxic activity [8,9,43,47,48,49]. As bufadienolides are pharmacologically highly active, depending on the type of compound and its dose, they might cause significant or even toxic effects for humans [50]. Because of that, it was very important to determine whether the bufadienolides contained in *Kalanchoe* juices are capable of passing through the skin to the circulatory system. The interest in the use of plant extracts from *K. blossfeldiana* and *K. pinnata* in dermatology and cosmetics is significant and caused by the high content of anti-aging polyphenolic compounds [10,11,33]. However, the use of *Kalanchoe* species that may contain bufadienolides should be subject to additional analysis regarding bufadienolide penetration through the skin. On the other hand, other steroidal compounds like saponins have beneficial activity on the skin’s surface, which should also be noted and taken under consideration [51].

Thus, this study shows, for the first time, that using the juices of plants from *Kalanchoe* spp. in preparations applied to the skin indicates that the bufadienolide compounds presented in these juices have no ability to penetrate the skin membranes. These data also confirm the potential of the ethnopharmacological use of *Kalanchoe* spp. juices in the treatment of dermatoses. It should be noted, however, that the research was conducted in vitro. The safety of applying juices and/or extracts on living organisms requires further research. Numerous studies analyzing xenobiotics have shown their higher penetration through the skin in the presence of sorption promoters naturally observed in plant extracts like terpenes [52] or added to skin-care products [53]. Therefore, when preparing a formulation for skin application with *Kalanchoe* juices or extracts that may contain bufadienolide compounds, their penetration should be assessed in detail.

## 4. Materials and Methods

### 4.1. Materials

Methanol, chloric acid, sulfuric acid, phosphoric acid, DMSO (dimethyl sulfoxide), and acetonitrile HPLC grade were purchased from Merck (Darmstadt, Germany). ABTS, bovine serum albumin (BSA), DPPH (2,2-diphenyl-1-picrylhydrazyl), ascorbic acid, TPTZ (2,4,6-tri(2-pyridyl)-s-triazine), hyaluronidase from bovine testes, FeCl_3_ × 6H_2_O, 20 mM phosphate buffer (pH 5.35), NaCl, hyaluronic acid, L-DOPA, tyrosinase from mushrooms, kojic acid, oleanolic acid monopotassium phosphate, and ammonium heptamolybdate were sourced from the Sigma Chemical Co. (St. Louis, MO, USA).

Ultrapure water was obtained in-house with a purification system (Milli-Q-Simplicity-185, Millipore Corp., Merck Millipore, Burlington, MA, USA).

### 4.2. Plant Material

Fresh leaves from three *Kalanchoe* species, *K. blossfeldiana*, *K. daigremontiana*, and *K. pinnata*, were sourced from a commercial garden (Garden Centre Justyna, Gdansk, Poland) and identified and stored in an Herbarium of the Medical University of Gdansk (GDMA Herbarium, Poland). Voucher specimens were 21,761–21,763 for *K. daigremontiana*, *K. pinnata*, and *K. blossfeldiana*, respectively. Healthy leaves (40 g of each species) were collected and cleaned with deionized water. Fresh leaves were cut, crushed, and filtered using a filter disc (390 grade, Filtrak). Fresh juices were obtained in volumes of 8.25 mL, 8.3 mL, and 27 mL for *K. blossfeldiana*, *K. daigremontiana*, and *K. pinnata*, respectively. After vacuum evaporation (40 °C), the juices were lyophilized. Until analysis, obtained powders were stored at −20 °C.

### 4.3. Qualitative and Semi-Quantitative Analyses of Bufadienolides

Lyophilized juices obtained from the leaves of *K. blossfeldiana*, *K. daigremontiana*, and *K. pinnata* were dissolved in methanol, sonicated, and filtered by syringeless filters (Whatman, Mini-UniPrep, Sigma-Aldrich). The final concentration of samples was 10 mg/mL of the dry mass. The juice samples were analyzed qualitatively and semi-quantitatively, similarly to previously described methods [9]. Briefly, the samples were run on a Dionex UltiMate 3000RS UHPLC system with a charged aerosol detector (CAD) connected to a high-resolution quadrupole time-of-flight mass spectrometer (QTOF-MS, Impact II HD, Bruker Daltonik GmbH, Billerica, MA, USA). The samples were separated with an Acquity UPLC HSS C18 column (150 × 2.1 mm, 1.8 μm, Waters) at a temperature of 55 °C. The mobile phases were water (solvent A) and acetonitrile (solvent B), both acidified with 0.1% formic acid. The separation gradient started with 20% of B from 0 to 1 min, then progressed to 39% B over 19.5 min. The sample injection volume was 5.0 μL, and the flow rate was 500 μL/min. The outflow from the column was split in a 3:1 proportion between CAD and QTOF-MS using a static splitter.

The electrospray ionization (ESI) analyses were conducted in positive ion mode, with a mass scan range of *m*/*z* 100–1500 and 5 Hz acquisition rate. The ion source operated with a capillary voltage of 4.5 kV, dry gas (N_2_) flow of 6.0 L/min, dry temperature of 200 °C, and nebulizer gas (N_2_) pressure of 2.7 bar. The spray chamber of CAD was kept at 35 °C, and a filter constant of 1 s and power function of 1.2 were applied during the data acquisition at a 10 Hz rate. Signals obtained from a charged aerosol detector (CAD, Thermo Corona Veo RS, Waltham, MA, USA) were used to evaluate the concentrations of bufadienolides. The CAD response was calibrated using 3β-(O-α-L-rhamnopyranosyl)-5β,11α,14β,19-tetrahydroxybufa-20,22-diene (compound **1** in Table 1) and 1,3,5-bersaldegenin-orthoacetate (compound **12**), in concentrations between 0.15 and 150 ng/μL (10 calibration points) using a series of dilutions from 1.0 mg/mL stock solutions. The contents of the two reference compounds in the investigated juices were quantitated directly from the obtained calibration curves. The concentrations of the remaining bufadienolides were estimated as weighted average responses of the two calibration references. Weights were calculated using RT differences. The identification of compounds was based on accurate mass measurements and comparisons with reference standards and MS/MS spectral data obtained previously [23]. The data were processed using DataAnalysis 4.3 (Bruker Daltonik GmbH). Identification work was also aided by SIRIUS software (ver. 4.6) for ranking ion isotope patterns [54]. The results are presented in Table 1, as well as Figure S1A–F (Supplementary Materials).

### 4.4. Bufadienolides’ Permeability Through the Strat-M Membrane

The studies were performed according to the method developed by our team previously [15,55] with some modifications. The Strat-M membrane (Merck KGaA, Darmstadt, Germany) was utilized for transdermal diffusion testing. The Strat-M membrane is a synthetic model of human skin widely used in the pharmaceutical and cosmetic industries, particularly for transdermal xenobiotics delivery and skin permeability testing. Strat-M membranes mimic the barrier properties of human skin, which makes them ideal for assessing active substances’ permeability and absorption and testing the effects of topical formulations. It provides a non-animal testing option, supporting ethical research and reducing the need for animal or ex vivo human experimentation in skin permeability studies. The membranes offer consistent performance across different experiments, making them highly reliable for research that requires reproducibility. Strat-M can be used in a wide range of applications, such as drug development, transdermal permeation studies, and cosmetic and medical device testing. Due to their reliability, Strat-M membranes are recognized and accepted by regulatory agencies like the FDA for use in testing skin permeability, which helps streamline the approval process for new products. On the other hand, Strat-M membranes are primarily designed to simulate the skin barrier for testing purposes and may not mimic other complex skin functions, such as immune responses and sweat gland activity. Also, they may not replicate all biological aspects of real skin. For instance, they lack living cells and physiological functions, which could limit the full accuracy of some types of skin˗related tests [56,57,58,59]. Skin penetration and skin permeation tests conducted in vivo or ex vivo may be subject to significant statistical error resulting from the condition of the skin used for testing. This applies not only to the age of the donor, but also to the physiological condition of the individual, as well as to gender and concomitant diseases that may affect the permeability of natural skin. Overall, human skin varies greatly among individuals (e.g., age, health condition of the donor, location on the body), and Strat-M is a standard model that might not account for this variability in some cases [58].

The juices dissolved in water were applied as an infinite dose at 5 mg/mL concentration. The pure water was applied to the skin as a blank control. The membrane diffusion area of 0.65 cm^2^ was placed in the flow-through Bronaugh diffusion cell apparatus. The analyzed solutions of the juices were applied on the upper surface of the membrane and left for 24 h in constant contact with acceptor fluid (saline solution, 20 mL with 0.005% sodium azide) that was thermostated and circulated with a constant rate of 10 mL/h. Acceptor fluid ensured the sink condition. The chamber system was incubated at 37 °C ± 0.5 °C. The temperature of the membrane was 32 °C. The permeant from the skin membrane was taken to the analysis after 2, 4, 6, 8, and 24 h according to the methods described in Section 4.3.

### 4.5. Antioxidant and Reduction Power Assays

The antioxidant reduction power capabilities of the *Kalanchoe* L. juices were estimated spectrophotometrically in a 96-well microplate reader (Epoch, BioTek System, Winooski, VT, USA). In the antioxidant assays, two tests were utilized: DPPH and ABTS. An evaluation of the reduction properties of the tested juices was estimated using FRAP (ferric reduction power) and molybdenum reduction power. In each test, ascorbic acid was used as the standard. The IC_50_ values of the juices were calculated using the GraphPad Prism 9 software (version 9.0.0, GraphPad Software, San Diego, CA, USA). The analyses were performed in triplicate, with three replications (*n* = 9).

#### 4.5.1. DPPH Assays

The radical scavenging activity of the juices from the *Kalanchoe* spp. species was determined by the DPPH method described by Kwon et al. [60]. Approximately 50 µL of different concentrations of the juices were dissolved in water and mixed with 100 µL of 0.06 mM DPPH (methanolic solution). The samples were kept in the dark for 30 min incubation (room temperature). Afterward, the absorbance was analyzed at λ = 517 nm. DPPH solution with water was used as a control.

#### 4.5.2. ABTS Assays

The radical scavenging activity of the juices from the *Kalanchoe* spp. species was determined by the ABTS method described by Kwon et al. [60]. Approximately 50 µL of different concentrations of the juices were dissolved in water and mixed with 170 µL of ABTS solution. After 10 min of incubation at 30 °C, the absorbance was analyzed at λ = 750 nm. ABTS solution with water was used as a control.

#### 4.5.3. FRAP Assay

The reducing ability of the juices from *Kalanchoe* spp. was determined with the FRAP test, based on the reduction of Fe^+3^ to Fe^+2^ [61]. Approximately 30 µL of serial dilutions of the juices or standard substance were mixed with 170 µL of the freshly prepared reaction mixture. After 20 min of incubation (room temperature), the absorbance was measured at 593 nm. The percentage of reduced iron ions was read from the calibration curve plotted for ascorbic acid (1–1000 µg/mL).

#### 4.5.4. Molybdenum Reducing Power

The reducing power of the juices was determined using the phosphomolybdenum method of Prieto et al. [62] with slight modifications. The sample and reaction mixture were incubated at 90 °C for 90 min, and after cooling, the absorbance at 700 nm was determined.

### 4.6. Enzyme Inhibition

The capabilities of the *Kalanchoe* L. juices to inhibit tyrosinase and hyaluronidase were estimated spectrophotometrically in a 96-well microplate reader (Epoch, BioTek System, Winooski, VT, USA). In each analysis, blank probes were prepared. The dose-dependent course of inhibition was analyzed. The IC_50_ value, a concentration of the juices at 50% tyrosinase and hyaluronidase inhibition, respectively, was calculated using GraphPad Prism (version 9.0.0, GraphPad Software, San Diego, CA, USA). Three independent experiments repeated in triplicate were performed.

#### 4.6.1. Tyrosinase Assay

Tyrosinase inhibitory activity was analyzed with the method described by Yagi et al. [63] and modified by Hering et al. [64]. L-DOPA was used as a substrate and kojic acid as a standard substance. The reaction mixture comprised phosphate buffer (0.175 mM, pH 6.8), tyrosinase (120 U), and different concentrations of juices. After 15 min of preincubation, L-DOPA (10 mM) was added. Product formation changes were recorded at λ = 475 nm for 20 min (Epoch BioTek System, Winooski, VT, USA).

#### 4.6.2. Hyaluronidase Assay

The hyaluronidase inhibitory activity of each juice was investigated using the method previously described by Kaessler et al. [65] with slight modifications [64]. The reaction mixture was incubated for 10 min in a water bath with different concentrations of juices. Hyaluronic acid (phosphate buffer 300 mM, pH 5.35) was then added to start the reaction. The undigested substrate was precipitated during the ten-minute incubation with bovine acid albumin, and the measurement was performed at λ = 600 nm. Oleanolic acid was used as a standard.

### 4.7. SPF Factor Determination

Methanol aliquots of all the juices were prepared in the 62.5–1000 μg/mL concentration range and analyzed spectrophotometrically (Epoch, BioTek System, Winooski, VT, USA). The dilutions were placed in a quartz cuvette (1 cm length), and the absorbance spectra were recorded in triplicate (λ = 280–500 nm, including 5 nm intervals). Methanol was used as a blank. Sun photoprotection factor in vitro was estimated according to the Sayre method, modified by Mansur et al. [66,67]. SPF in vitro was calculated according to the following equation:

SPF in vitro = CF × ∑ 320 290 EE(λ) × I(λ) × A(λ)

where:

EE(λ): the erythemal effect spectrum at wavelength λ;

I(λ): the solar intensity spectrum at wavelength λ, and the values of EE (λ) × I were constant;

A(λ): the absorbance of the juice solution determined by UV spectrophotometry at a wavelength (λ);

CF: the correction factor.

### 4.8. Statistical Analysis

Obtained data are presented as mean ± standard deviation (SD) and resulted from three experiments with three repetitions (*n* = 9). The statistically significant differences among groups were analyzed with one-way ANOVA with Tuckey’s post hoc test, *p* < 0.05. The statistically significant difference between the results of the juice sample and the standard was assessed with Student’s *t*-test, *p* < 0.05.

## 5. Conclusions

The present work is the first study on the permeation of bufadienolide compounds contained in *Kalanchoe* juices through the skin membrane. The results indicate that the plant juices can be for external uses, especially as antiaging agents, and can enhance skin function by direct influence on the enzymes responsible for ECM degradation and inflammatory processes. Also, antioxidant potential and sun protection properties, especially in the case of *K. blossfeldiana* and *K. pinnata* juices, may be useful in fighting with free radicals and oxidative stress generated in the skin. All these properties of *Kalanchoe* juices indicate their application potential in antiaging cosmetic products. However, further in vivo tests are required.

## Figures and Tables

**Table 2:** DPPH and ABTS scavenging, as well as ferric and molybdenum reduction power assays, exhibited as IC_50_ values with standard deviation (±SD), resulting from obtained juices of leaves from different species of *Kalanchoe* and ascorbic acid as a standard.

		IC_50_ µg/mL		
	*K. blossfeldiana*	*K. daigremontiana*	*K. pinnata*	Ascorbic Acid
DPPH	50.7 ± 0.77 *	496.13 ± 1.74 *	61.1 ± 0.89 *	12.12 ± 0.18
ABTS	18.8 ± 0.21 *	559.75 ± 5.75 *	28.27 ± 0.73 *	3.25 ± 0.15
Ferric reduction power	123.19 ± 3.72 *	561.36 ± 2.91 *	99.03 ± 1.75 *	5.75 ± 0.55
Molybdenum reduction power	2183.42 ± 12.52 *	NR	1148.86 ± 15.46 *	22.12 ± 0.35

NR—not reached. The results are presented as mean values with standard deviation (±SD) obtained from three experiments in three repetitions (*n* = 9). The statistically significant differences between IC_50_ of the *Kalanchoe* spp. and the standard are marked with “*” (Student’s *t*-test, *p* < 0.05). *K. blossfeldiana*—*Kalanchoe blossfeldiana*; *K. daigremontiana*—*Kalanchoe daigremontiana*; *K. pinnata*—*Kalanchoe pinnata*.

**Table 3:** The IC_50_ values resulted from inhibiting ECM enzymes by the tested juices of leaves obtained from different species of *Kalanchoe*.

	IC_50_ [µg/mL]				
	*K. blossfeldiana*	*K. daigremontiana*	*K. pinnata*	Kojic Acid	Oleanolic Acid
Tyrosinase	317.17 ± 6.94 *	457.12 ± 5.33 *	206.13 ± 7.4 *	22.75 ± 0.15	n.t.
Hyaluronidase	251.5 ± 4.28 *	413.43 ± 5.71 *	116.15 ± 5.15 *	n.t.	50.12 ± 2.15

n.t.—not tested. The data were obtained from three experiments with three repetitions (*n* = 9). Mean ± SD. All the IC_50_ values were statistically significantly different (Student’s *t*-test, “*” *p* < 0.05) in comparison to the IC_50_ of the standards (oleanolic acid or kojic acid).

**Table 4:** SPF in vitro estimated for tested juices of leaves from *K. blossfeldiana*, *K. daigremontiana*, and *K. pinnata*.

	*K. blossfeldiana*	*K. daigremontiana*	*K. pinnata*
SPF in vitro	9.2 ± 0.61	6.0 ± 0.77	9.6 ± 0.53

Results are obtained from the juices’ concentration of 1 mg/mL (methanol dilution) from three independent analyses and presented as mean ± SD.

## Data Availability

All data are included in the manuscript.

## Supplementary Materials

The following supporting information can be downloaded at https://www.mdpi.com/article/10.3390/molecules30040802/s1: Figure S1A: MS2 spectrum (collision energy 34.6 eV) of the compound B at RT 2.7 min, tentative identification: bryophyllin B. The ion at *m*/*z* 413.1959 results from the loss of acetate residue and the adjacent hydroxyl group; Figure S1B: MS2 spectrum (collision energy 33.8 eV) of the compound D at RT 3.7 min. Tentative identification: bersaldegenin acetate isomer. The loss of the acetate residue and the adjacent aldehyde group at *m*/*z* 397.2030 has similar relative intensity as for analogous loss in the fragmentation of compound B; Figure S1C: MS2 spectrum (collision energy 33.7 eV) of the compound F at RT 4.9 min, identified as bryophyllin A with authentic standard. The neutral losses of both 104.4 and 78.03 indicate the presence of the orthoacetate group; Figure S1D: MS2 spectrum (collision energy 33.8 eV) of the compound G at RT 5.6 min, tentatively identified as bersaldegenin acetate isomer. The loss of 78.03 Da with much lower relative intensity than analogous losses in fragmentations of compounds B and D; Figure S1E: MS2 spectrum (collision energy 34.4 eV) of the compound K at RT 8.3 min, identified as daigremontianin with authentic standard. Similar to fragmentations of bryophyllin A (compound F), neutral losses of 104.04 and 78.03 indicate the presence of the orthoacetate group; and Figure S1F: MS2 spectrum (collision energy 32.9 eV) of the compound K at RT 13.9, identified as bersaldegenin-1,3,5-orthoacetate with authentic standard. As previously shown fragmentation spectra of orthoacetates, it features visible ions resulting from the neutral losses of 104.04 and 78.03 Da.
