# Supplementary material for: Bufadienolide Penetration Through the Skin Membrane and Antiaging Properties of Kalanchoe spp. Juices in Dermal Applications
Source: Molecules. 2025 Feb 9;30(4):802. doi: 10.3390/molecules30040802 (PMC11858577; doi:10.3390/molecules30040802)
Supplement: Supplementary file 1 [file molecules-30-00802-s001.zip › molecules-3385765-supplementary.pdf]

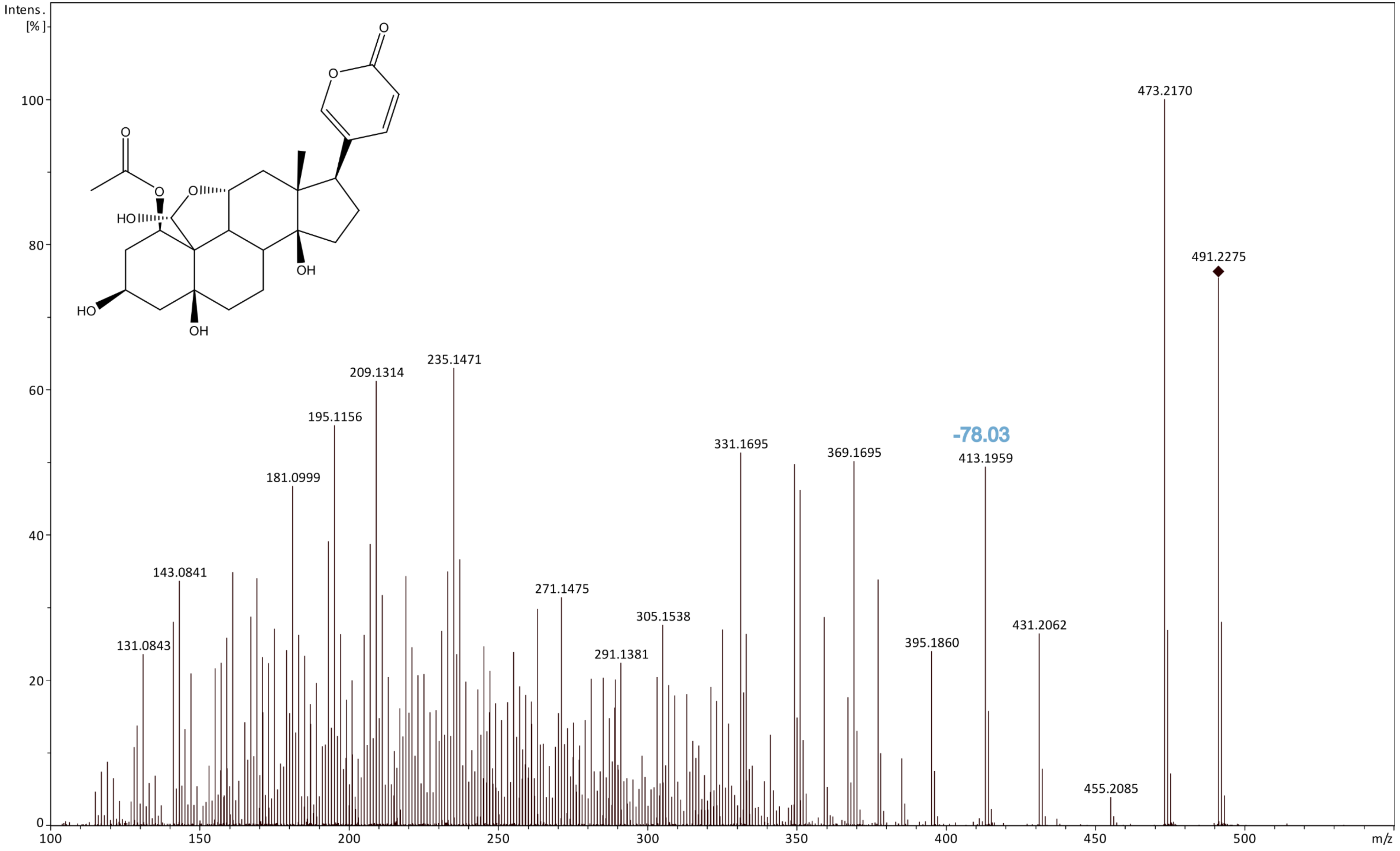

Figure S1A. MS2 spectrum (collision energy 34.6 eV) of the compound B at RT 2.7 min, tentative identification: bryophyllin B. The ion at *m/z* 413.1959 results from the loss of acetate residue and the adjacent hydroxyl group.

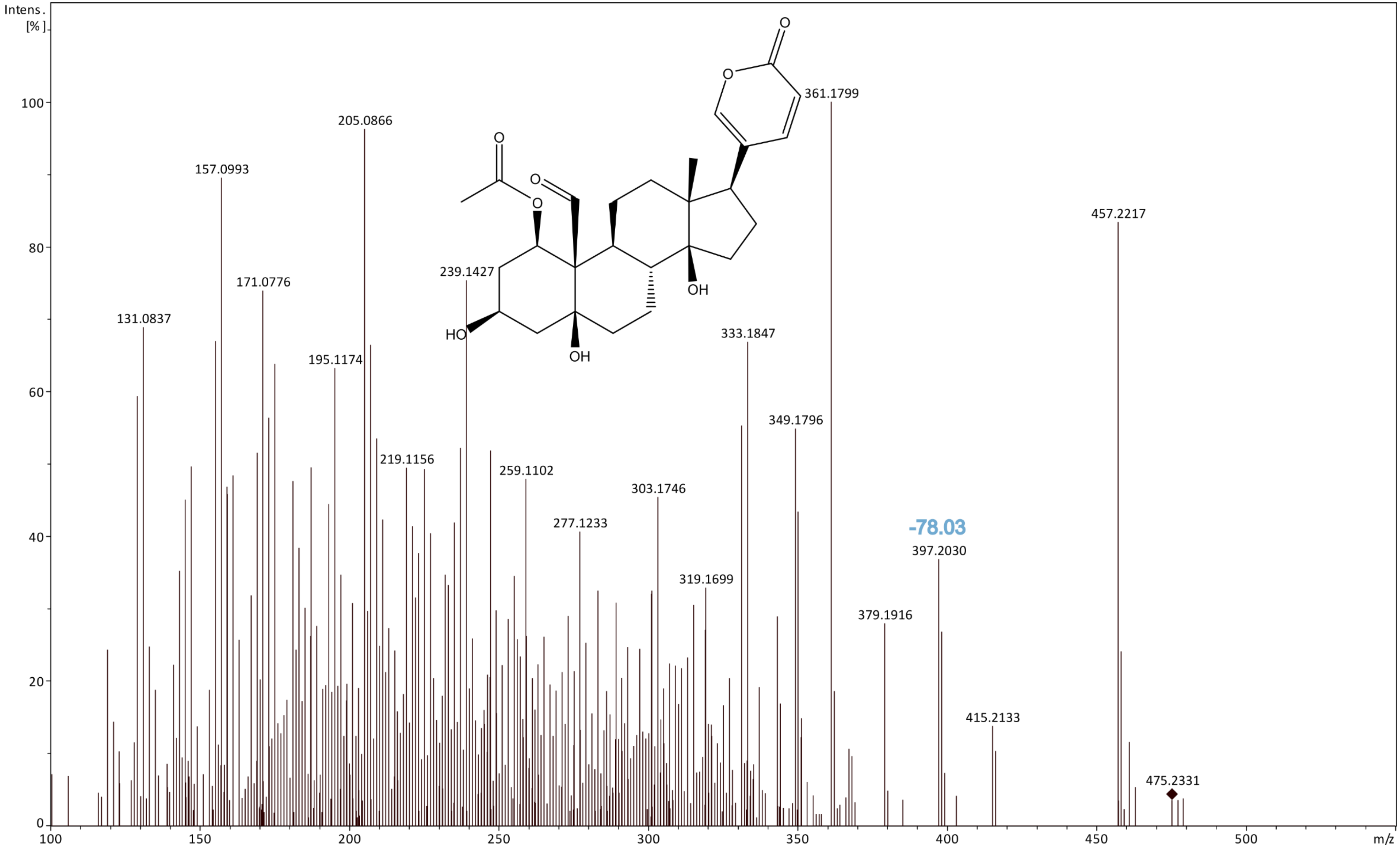

Figure S1B. MS2 spectrum (collision energy 33.8 eV) of the compound D at RT 3.7 min. Tentative identification: bersaldegenin acetate isomer. The loss of the acetate residue and the adjacent aldehyde group at *m/z* 397.2030 has similar relative intensity as for analogous loss in the fragmentation of compound B.

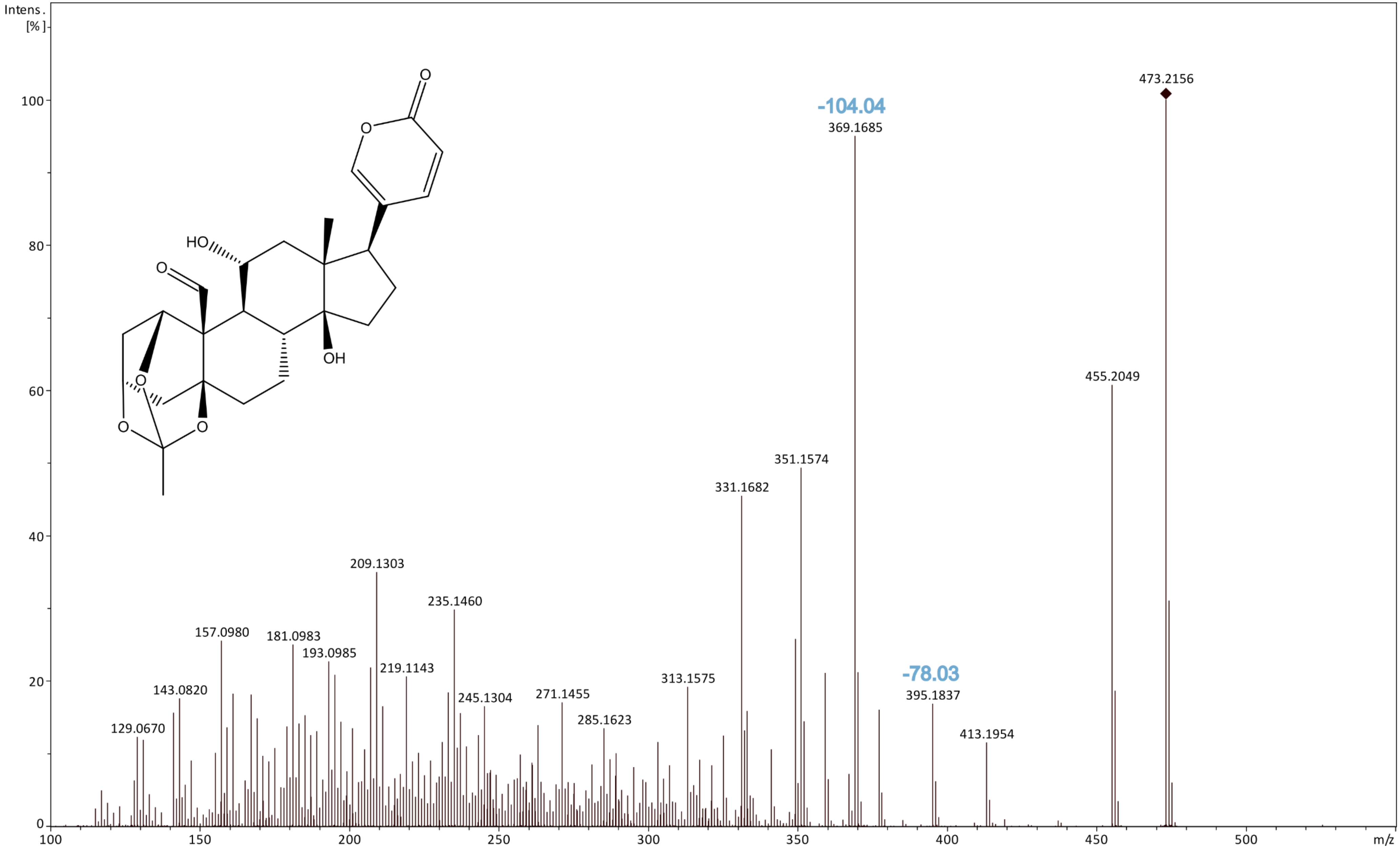

Figure S1C. MS2 spectrum (collision energy 33.7 eV) of the compound F at RT 4.9 min, identified as bryophyllin A with authentic standard. The neutral losses of both 104.4 and 78.03 indicate the presence of the orthoacetate group.

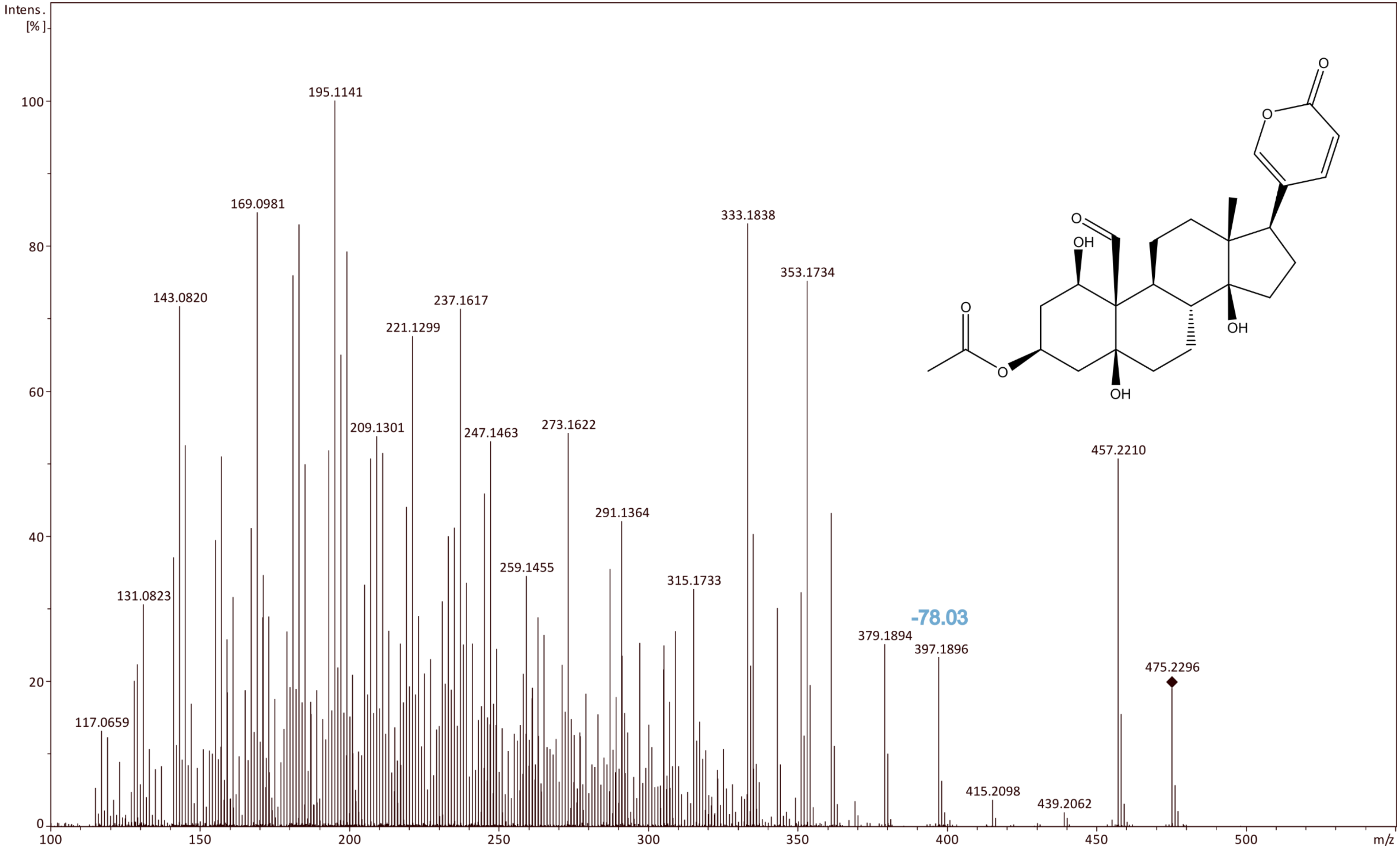

Figure S1D. MS2 spectrum (collision energy 33.8 eV) of the compound G at RT 5.6 min, tentatively identified as bersaldegenin acetate isomer. The loss of 78.03 Da with much lower relative intensity than analogous losses in fragmentations of compounds B and D.

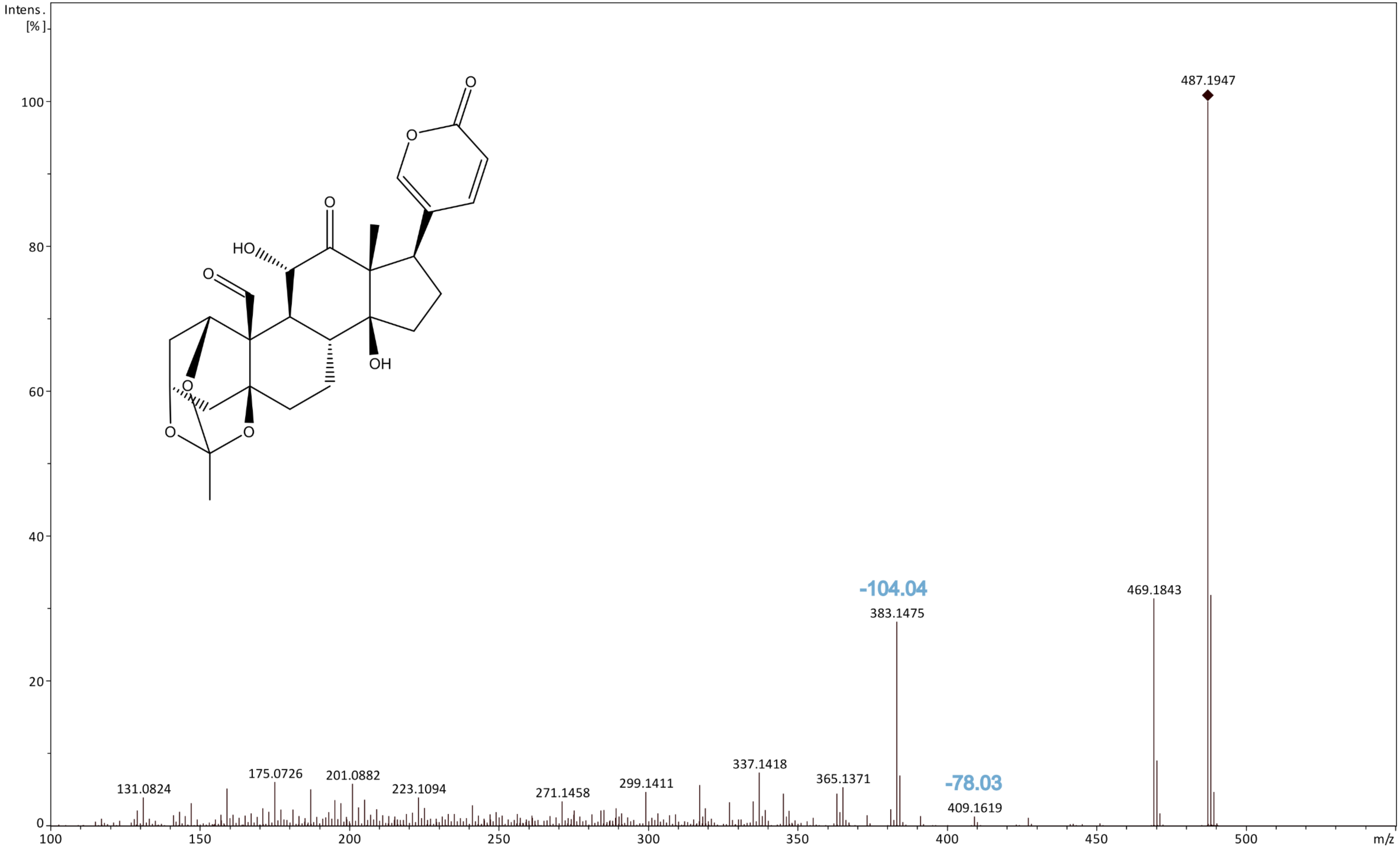

Figure S1E. MS2 spectrum (collision energy 34.4 eV) of the compound K at RT 8.3 min, identified as daigremontianin with authentic standard. Similar to fragmentations of bryophyllin A (compound F), neutral losses of 104.04 and 78.03 indicate the presence of the orthoacetate group.

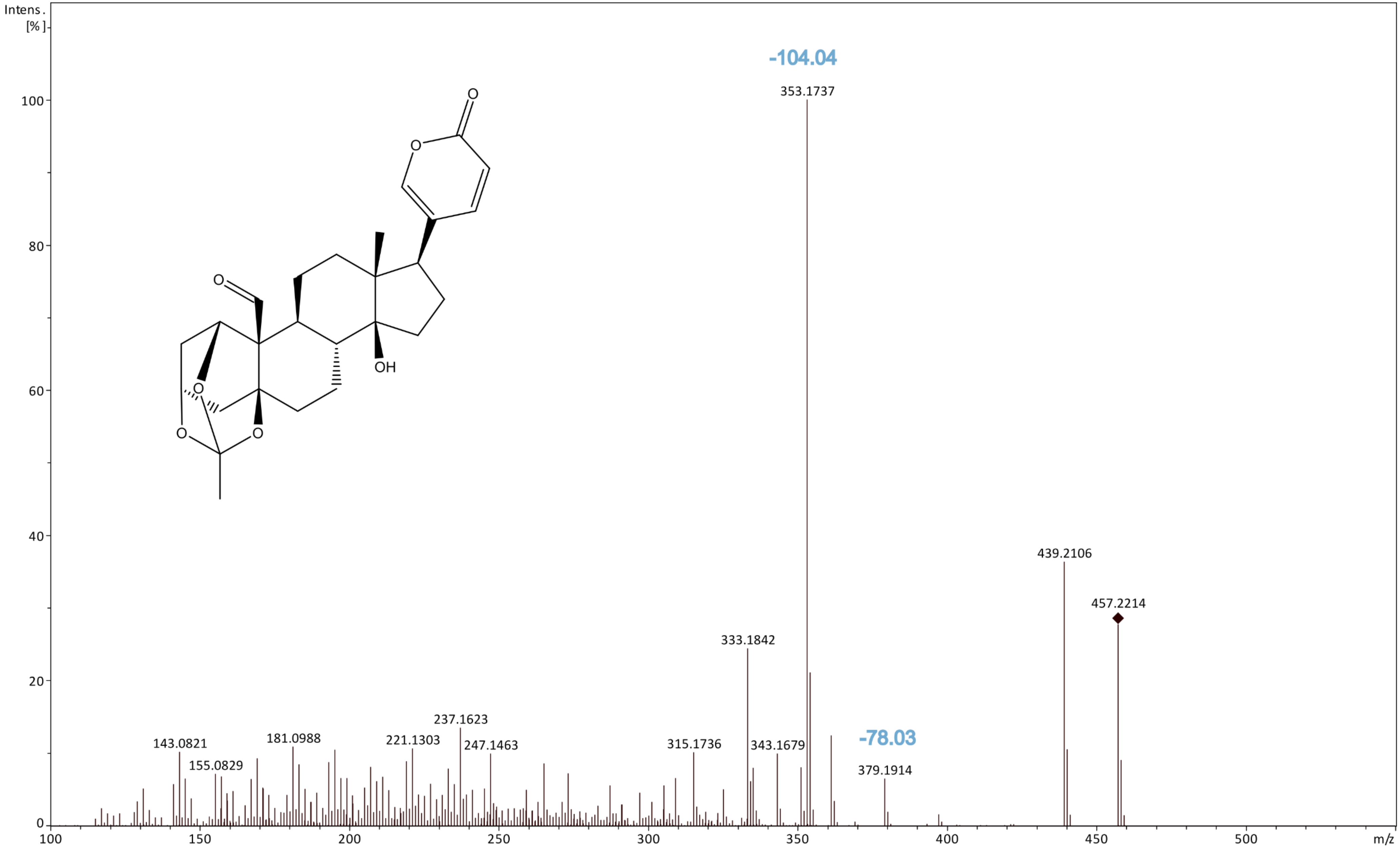

Figure S1F. MS2 spectrum (collision energy 32.9 eV) of the compound K at RT 13.9, identified as bersaldegenin-1,3,5-orthoacetate with authentic standard. As previously shown fragmentation spectra of orthoacetates, it features visible ions resulting from the neutral losses of 104.04 and 78.03 Da.
